# Transcriptome Sequencing Reveals the Traits of Spermatogenesis and Testicular Development in Large Yellow Croaker (*Larimichthys crocea*)

**DOI:** 10.3390/genes10120958

**Published:** 2019-11-21

**Authors:** Shengyu Luo, Xinming Gao, Jie Ding, Cheng Liu, Chen Du, Congcong Hou, Junquan Zhu, Bao Lou

**Affiliations:** 1 Key Laboratory of Applied Marine Biotechnology by the Ministry of Education, School of Marine Sciences, Ningbo University, Ningbo 315211, China; 1701091031@nbu.edu.cn (S.L.); 1511091840@nbu.edu.cn (X.G.); 1711091015@nbu.edu.cn (J.D.); 1801091036@nbu.edu.cn (C.L.); 1601091037@nbu.edu.cn (C.D.); houcongcong@nbu.edu.cn (C.H.); 2 Zhejiang Academy of Agricultural Sciences, Hangzhou 310021, China; Loubao@zaas.ac.cn

**Keywords:** *Larimichthys crocea*, RNA-Seq, testicular development, spermatogenesis

## Abstract

*Larimichthys crocea* is an economically important marine fish in China. To date, the molecular mechanisms underlying testicular development and spermatogenesis in *L. crocea* have not been thoroughly elucidated. In this study, we conducted a comparative transcriptome analysis between testes (TES) and pooled multiple tissues (PMT) (liver, spleen, heart, and kidney) from six male individuals. More than 54 million clean reads were yielded from TES and PMT libraries. After mapping to the draft genome of *L. crocea*, we acquired 25,787 genes from the transcriptome dataset. Expression analyses identified a total of 3853 differentially expressed genes (DEGs), including 2194 testes-biased genes (highly expressed in the TES) and 1659 somatic-biased genes (highly expressed in the PMT). The dataset was further annotated by blasting with multi-databases. Functional genes and enrichment pathways involved in spermatogenesis and testicular development were analyzed, such as the neuroactive ligand–receptor interaction pathway, gonadotropin-releasing hormone (GnRH) and mitogen-activated protein kinase (MAPK) signaling pathways, cell cycle pathway, and *dynein*, *kinesin*, *myosin*, *actin*, heat shock protein (*hsp*), synaptonemal complex protein 2 (*sycp2*), doublesex- and mab-3-related transcription factor 1 (*dmrt1*), spermatogenesis-associated genes (*spata*), DEAD-Box Helicases (*ddx*), tudor domain-containing protein (*tdrd*), and *piwi* genes. The candidate genes identified by this study lay the foundation for further studies into the molecular mechanisms underlying testicular development and spermatogenesis in *L. crocea*.

## 1. Introduction

Large yellow croaker (*Larimichthys crocea*) is an economically important maricultured species in southeastern China, and it has received considerable attention because of its high commercial and nutritional value. Due to breakthroughs in artificial propagation technology for *L. crocea* in 1990, its aquaculture production has increased rapidly. In 2017, the annual output of cultured *L. crocea* in China was approximately 176,600 tons, which ranked first among maricultured fishes [1]. However, with the rapid development of the culture industry, marine cage-cultured *L. crocea* have suffered from large-scale sexual precocity, which is extremely detrimental to the normal growth of fish [2]. An increasing number of *L. crocea* mature when small-sized, and once the fish are sexually mature, energy and nutrients are mostly shifted to the processes of gonad maturation or reproduction, and only a small amount remains for somatic growth; these changes eventually reduce the culture benefit of *L. crocea* [3] and hinder the conservation of its germplasm resources. Therefore, the molecular mechanisms underlying reproduction regulation must be investigated, and the genes involved in gametogenesis and gonadal development must be identified, to provide a theoretical reference for further studies on the above problems in *L. crocea*.

Directly correlated with sexual maturation and reproduction, in various animal species, including *L. crocea*, the testis is an essential basic component of the animal reproductive system and responsible for the production of male gametes via spermatogenesis [4]. During the process of spermatogenesis, diploid spermatogonia slowly evolve into many highly specialized spermatozoa through mitosis, meiosis, and spermiogenesis. Stringent spatial and temporal expression of genes during both transcriptional and translational processes are fundamental to ensure the precise processes of spermatogenesis [5]. Previous studies have focused on the reproductive and developmental biology of *L. crocea* based on anatomical/histological aspects [6,7], whereas few studies have focused on genes related to spermatogenesis and testicular development. To the best of our knowledge, only a small number of reproduction-related genes have been identified in *L. crocea* [8,9,10,11]. To further comprehensively explore the molecular mechanisms underlying spermatogenesis and testicular development in *L. crocea*, large-scale genetic resources must be identified and studied.

Recently, RNA-Seq technology has been widely used to explain biological processes in almost all organisms. Over the past three years, RNA-Seq analyses of reproductive tissues have been performed in several commercial teleosts, such as *Thalassoma bifasciatum* [12], *Takifugu rubripes* [13], and *Ictalurus punctatus* [14]. Numerous reproductive-related genes and signaling pathways have been screened, which has deepened our understanding of gonadal development and gametogenesis. In this study, we identified the genes and pathways potentially participating in testis development and spermatogenesis in *L. crocea*. This study could offer fundamental information for further research on the mechanisms underlying reproductive development in male *L. crocea*.

## 2. Materials and Methods

### 2.1. Ethics Statement

All fish handling and experimental procedures were approved by the Animal Care and Use Committee of Ningbo University. Prior to sampling, all fish were anesthetized with 0.05% MS-222 (3-Aminobenzoic acid ethyl ester methanesulfonate, Sigma, Saint Louis, MO, USA).

### 2.2. Sample Collection, Histological Identification, RNA Isolation, and cDNA Library Preparation

Healthy male fish (17 ± 0.8 cm, 59 ± 4.1 g) were commercially obtained and sampled from a local farm in May 2016. After 2 weeks of acclimatization in seawater (DO 8.6 ± 0.3 mg L^−1^, 23 ± 1 °C), fish were dissected on ice and the tissues (testis, liver, spleen, heart, and kidney) from 20 fish were removed, frozen in liquid nitrogen, and stored at −80 °C. Concurrently, a small part of the testis of each fish was used to perform histological analysis so that we could select individuals with testes at developmental stage IV, in which the process of spermatogenesis was active. The testicular developmental stages were identified based on histological features, as mentioned in a previous report [15]. After validating the testes development, 6 male individuals (stage IV) of the 20 fish were chosen for follow-up experiments. Total RNA was isolated with TRIzol reagent (Invitrogen, Shanghai, China) according to a standard protocol. RNA integrity, purity, and concentration were validated as described by Zhan [16] and Chen [17]. Only samples with an RNA Integrity Number (RIN) > 8.0 were used for RNA library construction. The MixS (minimum information about any (x) sequence) descriptors are presented in Table 1.

**Figure 1:**
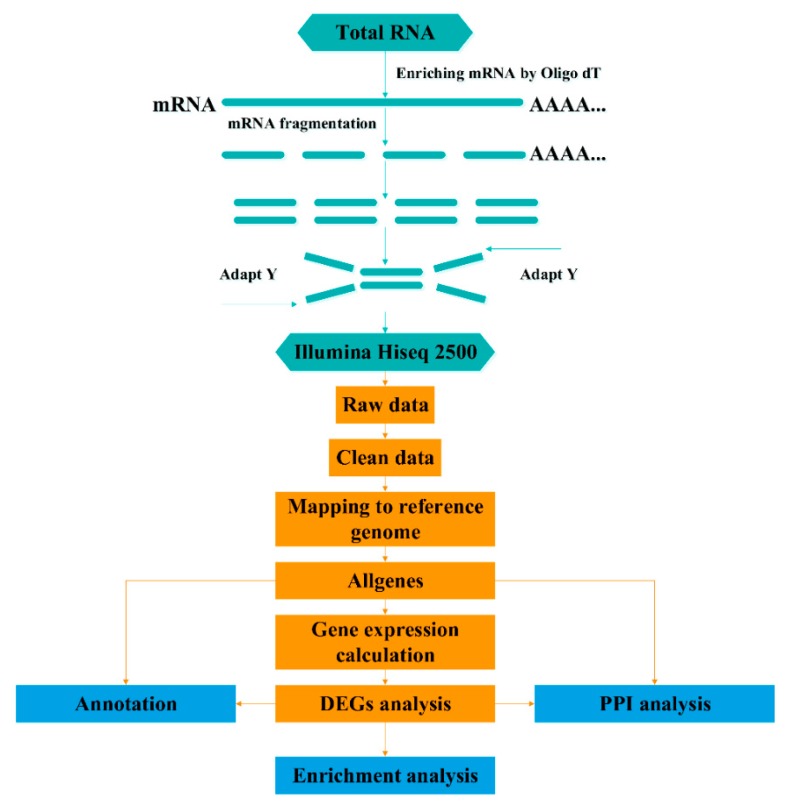
RNA-Sequencing data processing and analysis workflow.

To normalize individual differences, an equal amount of RNA isolated from the testes of the six male individuals were pooled and constructed a TES cDNA library. Similarly, an equal amount of RNA isolated from other somatic tissues (liver, spleen, heart, and kidney) were also pooled and constructed a PMT cDNA library. Sequencing library construction and Illumina sequencing were conducted at Beijing BioMarker Technologies (Beijing, China) following the manufacturer’s recommendations. The complete RNA-Seq process and bioinformatics data analysis workflow are presented in Figure 1.

### 2.3. Sequencing and Mapping to the Reference Genome

Clean data (clean reads) were obtained by removing reads containing adapters, ploy-N, and inferior reads from the raw data. Then, we used the *L. crocea* genome (accession number: PRJNA354443) as a reference for sequence alignment with the Tophat2 software. HTSeq v0.6.1 with −m union was used to count the number of reads mapped to each gene. For normalization, the count for each gene was divided by the number of fragments per kilobase of transcript sequence per million base pairs (FPKM) sequenced in each sample. FPKM were used to estimate the gene expression level.

### 2.4. Differential Expression Analysis

The differential expression analysis of two samples was performed using the EBseq R package. *p*-values were adjusted using the *Q* value [18], and *Q* value < 0.01 and |log_2_ (fold change)| ≥ 2 were set as the thresholds for significant differential expression.

### 2.5. Functional Annotation and Pathway Enrichment Analysis

The transcripts sequences in our study were aligned by BLASTx to the Nr, GO, Swiss-Prot, KEGG, and COG databases (*E*-value < 10^−5^) and aligned by BLASTn to the Nt database (*E*-value < 10^−5^). Candidate DEGs potentially associated with spermatogenesis and testicular development in *L. crocea* were identified by the combination of enrichment analyses, annotations, and manual literature searches.

### 2.6. Quantitative Real-Time PCR (qPCR) Validation

A total of 15 DEGs with significantly different expression in the TES and PMT were randomly selected for validation of the Illumina sequencing data via a qPCR (LightCycler 480 Roche) analysis. cDNA was synthesized using the ReverTra Ace qPCR RT Master Mix with gDNA Remover (TOYOBO BIOTECH CO., LTD. Shanghai, China) with the total RNA used in the RNA-Seq analysis. The primers designed for amplification were based on RNA-Seq data in our library and NCBI nucleotide databases. All primer sequences are listed in Table 2. The reaction was performed in a 20 μL reaction volume containing 4 μL cDNA, 10 μL 2×RealStar Green Fast Mixture (Genstar, China), 1 μL each primer (10 mM), and 4 μL nuclease-free water. β-actin was used as an internal control to normalize the gene expression level. The PCR conditions were as follows: 10 min at 95 °C, 40 cycles at 95 °C for 30 s, 60 °C for 30 s, 72 °C for 30 s, and followed by a cooling step at 4 °C. Every sample was amplified in triplicate to normalize the system and reduce pipetting errors. The 2^−ΔΔ*C*T^ method was used to analyze the relative expression level. Pearson’s correlation coefficient was used to assess the expression data consistency between the RNA-Seq and qPCR.

## 3. Results

### 3.1. Identification of Testes in Developmental Stage IV

**Figure 2:**
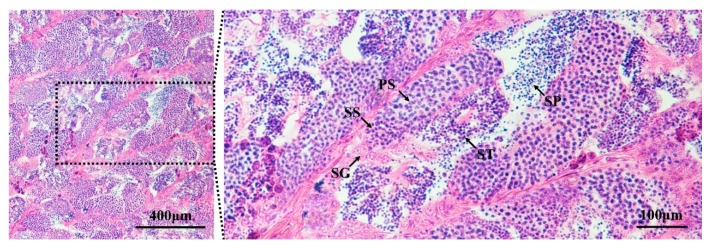
Developmental stage identification of testes by histological analysis. The results show that the testes were in developmental stage IV. The following germ cell populations were observed in the testes: SG, spermatogonia; PS, primary spermatocyte; SS, secondary spermatocyte; ST, spermatid; and SP, spermatozoon.

We successfully screened *L. crocea* individuals with testes in developmental stage IV. All germ cell populations from spermatogonia to spermatids were observed in these testes. In addition, many spermatozoa differentiated from spermatids were released into the lobular cavity (Figure 2).

### 3.2. Overview of the Transcriptome Profiles

An overview of the reads and quality filtering of the two libraries is presented in Table 3. The Illumina HiSeq 2500 platform yielded 26.2 million and 28.78 million clean reads from the TES (testes) library and the PMT (pooled multiple tissues) library, respectively; and 33,390,310 and 38,470,859 reads were mapped to the *L. crocea* genome, representing 63.72% and 66.82% of the clean reads from the two samples, respectively. The mapped reads represented nearly 70% of the *L. crocea* genome. The Q30 values (percentage of sequences with a sequencing error rate lower than 0.1%) in the two libraries were 85.81% and 86.27%, respectively, and the GC percentages of the libraries were almost 50%, indicating that the sequencing results were reliable (Table 3).

**Figure 3:**
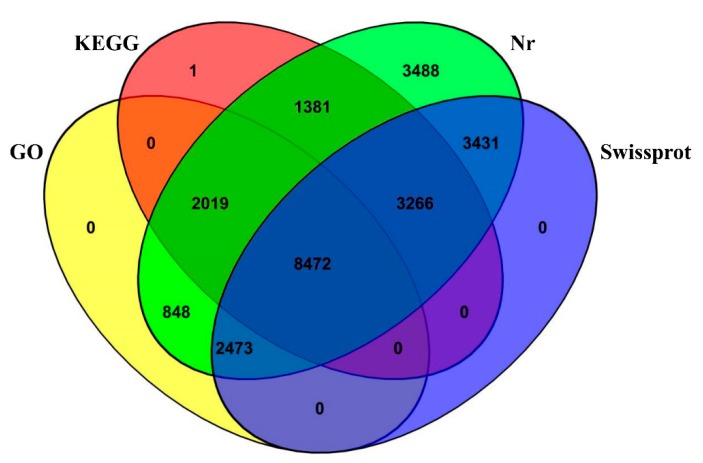
Venn diagram showing the annotation by four databases. For homology annotation, non-redundant sequences were searched using public databases, including the Nr, Swiss-Prot, KEGG, and GO databases.

**Figure 4:**
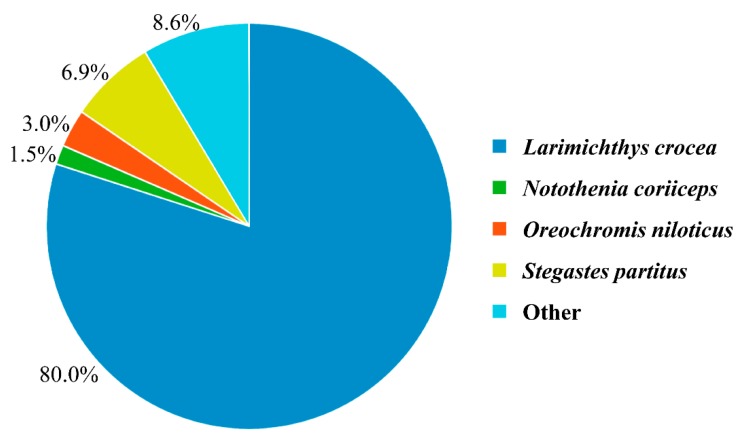
Species that match the annotated sequences of *L. crocea*. The species distribution is based on the results of the Nr annotation. The percentages of contigs that are homologous with other species are presented.

For an overview of the function of all genes from our dataset, the 25,787 *L. crocea* genes were annotated based on multiple databases using the BLASTx or BLASTn algorithm (*E*-value ≤10^−5^). A total of 25,384 (98.44%) genes were annotated, with 25,378 (98.41%) genes annotated in the Nr database, followed by the Swiss-Prot (17,642; 68.41%), KEGG (15,140; 58.71%), and GO (13,813; 53.57%) databases (Figure 3). To assess their evolutionary conservation, the 25,378 genes mapped to the Nr database were searched against the sequences of other species in the Nr database using the BLASTx algorithm. The results show the highest homology with *L. crocea* (80.0%) (Figure 4).

**Figure 5:**
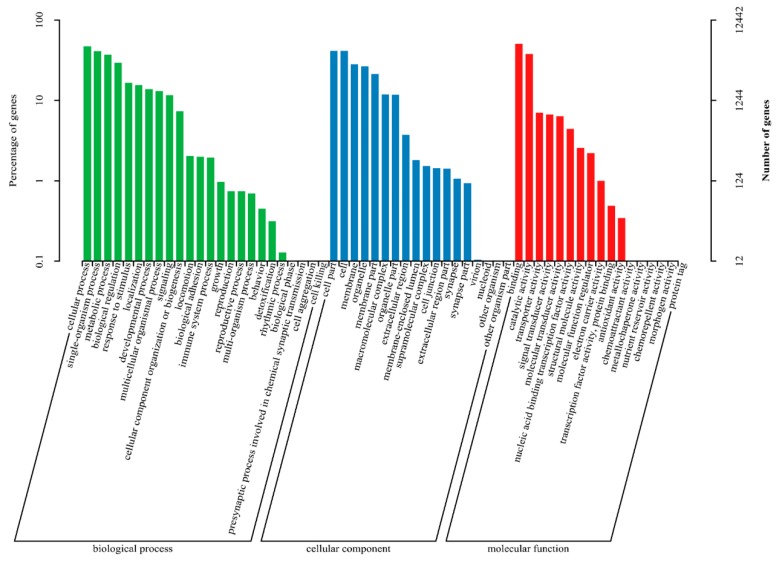
All genes GO cluster distribution. Genes were classified into three main categories: Biological process, cellular component, and molecular function.

According to the GO annotation result, 13,813 of all genes were mapped to the GO database and classified into three major functional categories (biological process, cellular component, and molecular function) and 59 subcategories. Amongst these genes, 10,237 genes (74.11%) were categorized into the “biological process” functional group; 7396 genes (53.54%) were categorized into the “cellular component” functional group; and 11,697 genes (84.68%) were categorized into the “molecular function” group (Figure 5).

### 3.3. Differentially Expressed Genes (DEGs) of the Two Libraries

**Figure 6:**
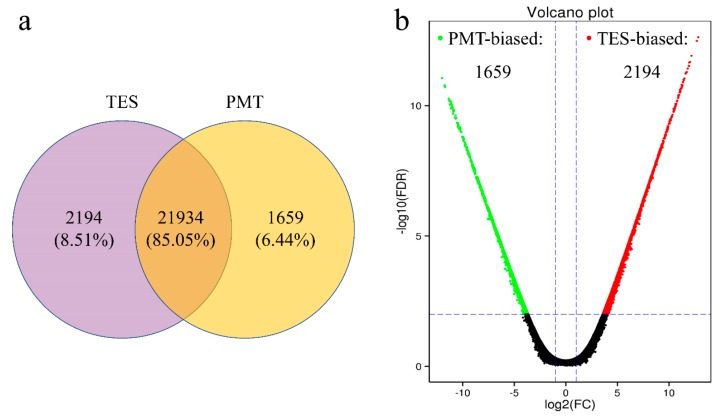
Comparative results of RNA-Seq and differentially expressed gene distributions between the TES and PMT. (**a**) Venn diagram showing genes expressed highly in the TES (light purple circle), expressed highly in the PMT (light yellow circle), and expressed without significant difference in both samples (intersection). (**b**) Volcano scatter plot of differentially expressed genes (TES vs. PMT). Red points represent the testes-biased genes with log_2_ (fold change) > 2 and *Q* value < 0.01, i.e., −log_10_ (*Q* value) ≥ 2.0. Green points represent somatic-biased genes with log_2_ (fold change) < −2 and *Q* value < 0.01, i.e., −log_10_ (*Q* value) ≥ 2. Black points represent genes with no significant differences. Fold change equal to normalized gene expression of the TES/normalized gene expression of the PMT.

**Figure 7:**
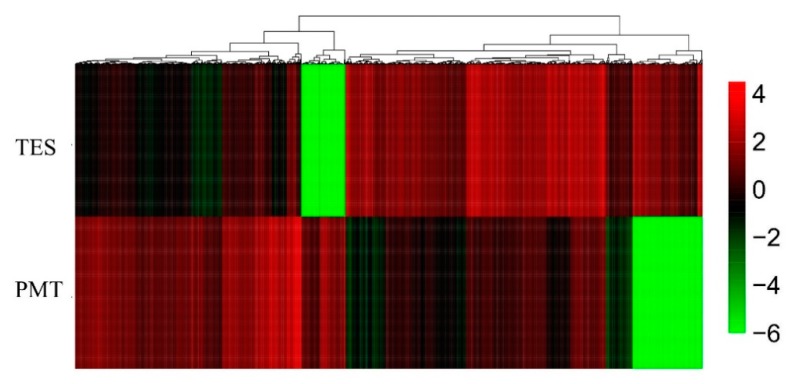
Hierarchical clustering of differentially expressed (DE) mRNAs among the TES and PMT. Heatmap of the count data for DE mRNA libraries for the DEGs identified between the TES and PMT.

A total of 21,934 genes did not show significant differences in expression in both groups, while 2194 genes (testes-biased genes) were highly expressed in the TES and 1659 genes (somatic-biased genes) were highly expressed in the PMT (Figure 6a,b). Based on the *Q* value < 0.01 and |log_2_ (fold change)| ≥ 2 threshold, 3853 DEGs were identified between the TES and PMT. Among the 3853 DEGs, 332 were novel genes. Detailed DEGs information is shown in Table S1. The expression of DEGs from the two samples clustered into two distinct groups based on hierarchical clustering (Figure 7).

### 3.4. DEGs Annotation and Pathway Analysis

**Figure 8:**
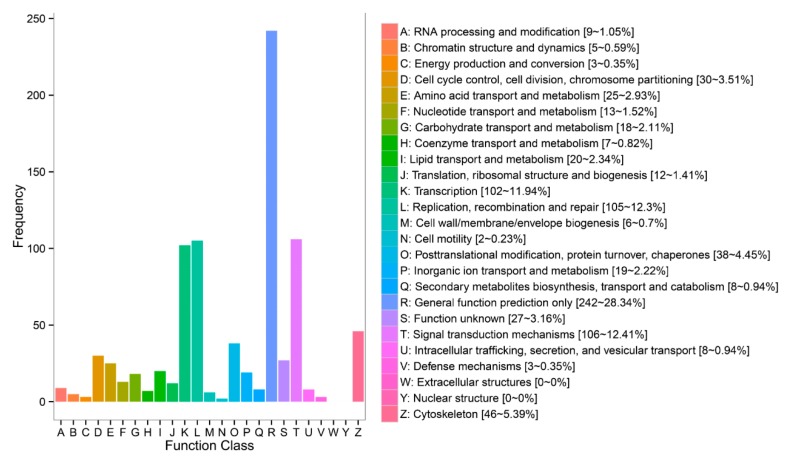
Clusters of orthologous groups (COG) classification; 541 testes-biased genes with Nr hits were grouped into 23 COG classifications.

Functional prediction and classification of the testes-biased gene sequences were achieved by searching against the COG database, and the DEGs were classified into 24 categories (Figure 8). The cluster “general function prediction only” was the major group (242, 28.34%), followed by “signal transduction mechanisms” (106, 12.41%); “replication, recombination, and repair” (105, 12.30%); “transcription” (102, 11.94%); “cytoskeleton” (46, 5.39%); “posttranslational modification, protein turnover, chaperones” (38, 4.45%); and “cell cycle control, cell division, chromosome partitioning” (30, 3.51%). No DEGs were sorted into the “extracellular structures” and “nuclear structure” categories (Figure 8).

**Figure 9:**
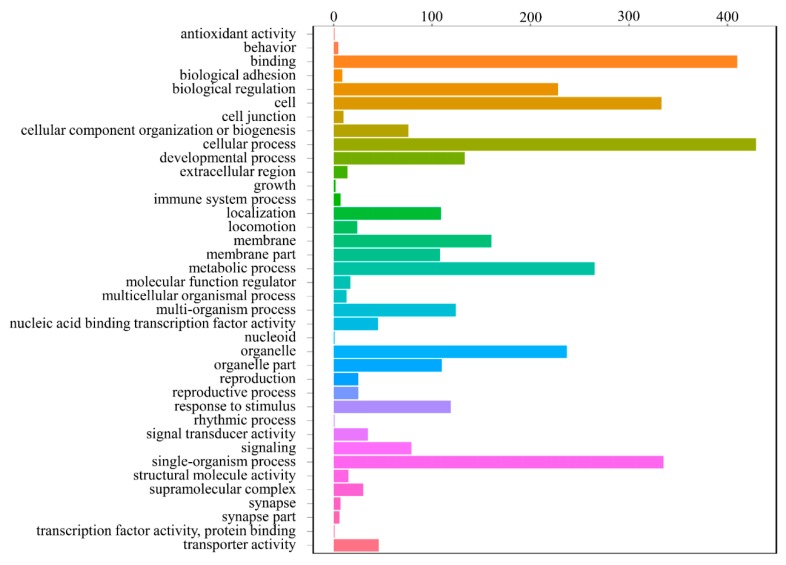
Gene ontology (GO) classifications of testes-biased genes. The X-axis presents the number of genes; the Y-axis presents 38 function subcategories.

According to the GO analysis, 776 testes-biased genes were mapped to the GO databases and classified into three major functional categories (biological process, cellular component, and molecular function) and 38 subcategories (Figure 9 and Table S2). Amongst these, 634 genes (81.70%) were categorized into the “biological process” functional group; 438 genes (56.44%) were categorized into the “cellular component” functional group; and 589 genes (75.90%) were categorized into the “molecular function” group (Table S2). Moreover, among the 38 significantly enriched GO subcategories (*Q* < 0.05), 19 were associated with “biological process”, 11 were associated with “cellular component”, and 8 were associated with “molecular function”. For biological processes, the most frequently occurring GO term was “cellular process”. For cellular components, the terms “cell” and “organelle” showed the highest frequency. In the category of molecular function, the term “binding” accounted for the largest proportion of annotations, followed by “transporter activity” (Table S2). Some of these enriched GO subcategories are potentially involved in spermatogenesis and testicular development, such as “reproduction”, “reproductive process”, “cellular process”, “biological regulation”, and “developmental process”.

In addition, 32 third-level GO functional categories closely related to spermatogenesis and testis development were significantly enriched from the present dataset with *KS* < 0.05 (Table 4). These categories covered multifaceted physiological and biological processes of spermatogenesis and testicular development and offered many candidate genes for further investigation.

**Figure 10:**
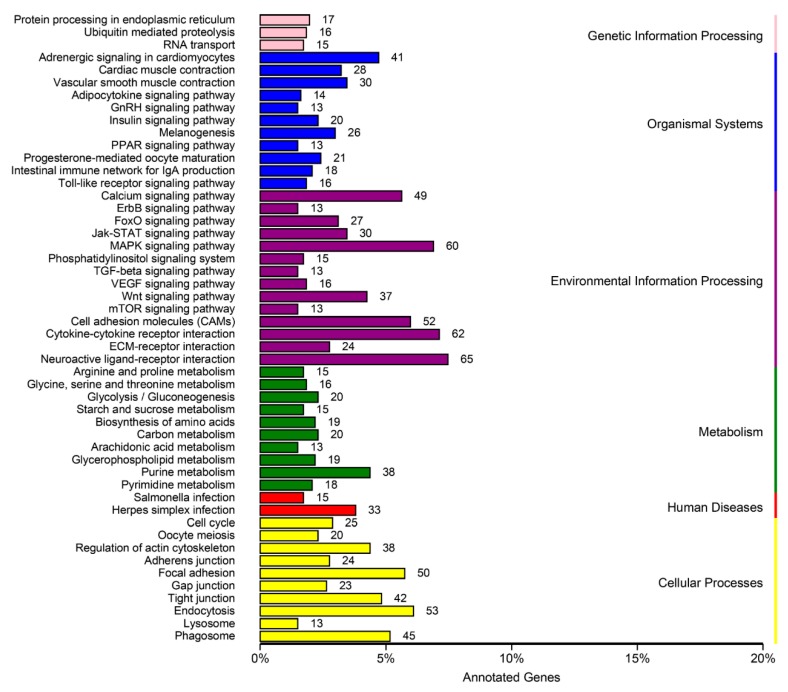
KEGG classifications of DEGs. The X-axis presents number and percentage of annotated DEGs; the Y-axis presents top 50 pathways.

To further identify molecular interaction networks within cells, the DEGs were mapped to the KEGG Pathway Tools and assigned to a total of 152 pathways (Table S3). The top 50 pathways are presented in Figure 10. Among them, several signaling pathways were identified and documented as essential in gonadal development and maturation, including the following pathways: Neuroactive ligand–receptor interaction pathway (65 genes), MAPK signaling pathway (60), focal adhesion (50), purine metabolism (38), regulation of actin cytoskeleton (38), cell cycle (25), adherens junction (24), oocyte meiosis (20), pyrimidine metabolism (18), pyrimidine metabolism (18), ubiquitin-mediated proteolysis (16), GnRH signaling (13), steroid hormone biosynthesis (12), spliceosome (5), mismatch repair (3), DNA replication (2), and RNA polymerase (2) (Figure 10).

**Figure 11:**
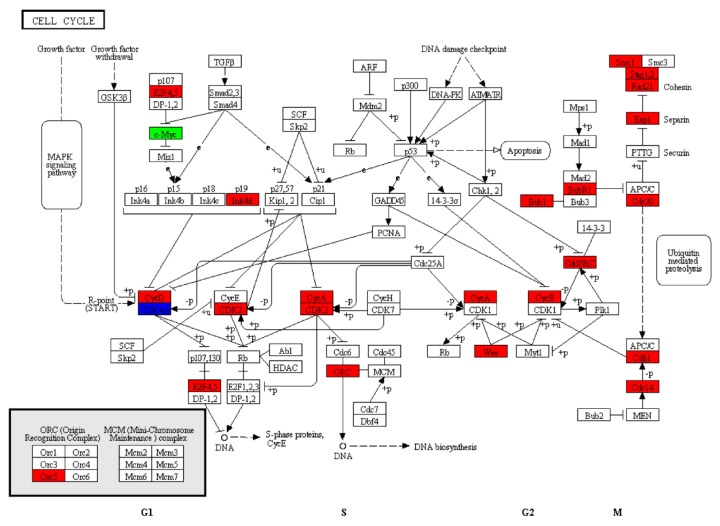
Cell cycle pathway. Green, significantly decreased expression; blue, proteins encoded by both up- and downregulated genes; red, significantly increased expression.

Based on the literature review results, the role of the cell cycle signaling pathway in spermatogenesis was highlighted. There were 23 significantly TES-biased genes (*cdc14a*, *cyclin b3*, *e2f5*, *cyclin d2*, *espl*, *fzr1*, *cdkn2d*, *bub1*, *bub1 beta*, *cyclin-a1*, *cyclin-b2-like*, *wee 2*, *cdk 2*, *orc5*, *rad21*, *cyclin b2*, *cdc20*, *cyclin-a2*, *cdk 4*, *stag1*, *cdc25-1-b*, *cdca20*, and *smc1b*) and 2 significantly PMT-biased genes (*cdk6* and *myc-2*) found in the cell cycle (Figure 11).

### 3.5. Validation of RNA-Seq Data by qPCR

**Figure 12:**
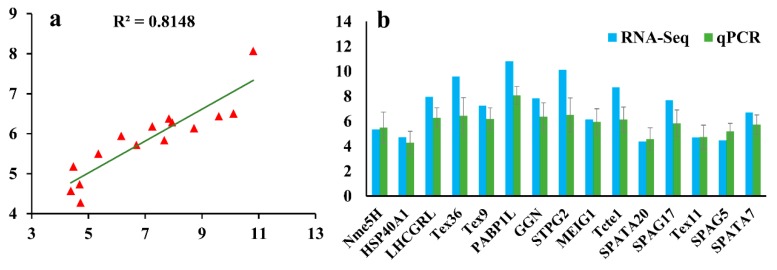
Validation of RNA-Seq data using qPCR. (**a**) Consistency of log_2_FoldChange (log_2_FC) between RNA-Seq (*X*-axis) and qPCR assay (*Y*-axis) is high (R^2^ = 0.814) based on the 15 selected genes. (**b**) Log_2_FC of the selected DEGs by qPCR compared with RNA-Seq data. The log_2_FC values of qPCR are shown as the means ± standard error (SE).

To verify the reliability of the transcriptome data, we measured the mRNA expression levels of 15 DEGs by qPCR. The results show that the data from the qPCR and the transcriptome sequencing tend to be consistent (Figure 12), and the Pearson’s correlation coefficient of 0.814 between the transcriptome and qPCR gene expression results confirmed the reproducibility and reliability of the Illumina sequencing.

## 4. Discussion

Although *L. crocea* is an economically important marine fish in China, comprehensive studies on its gonadal development and gametogenesis molecular mechanisms are lacking. Descriptive and quantitative RNA-Seq analyses are crucial for elucidating functional elements of a genome and uncovering the molecular constituents of cells and tissues. RNA-Seq technology has emerged as a powerful tool for studying the molecular mechanisms of certain biological processes in all organisms. To identify the genes and pathways related to spermatogenesis and testicular development of *L. crocea*, we performed, for the first time, a comparative transcriptome analysis between the testes (stage IV) and other somatic tissues. The testes in stage IV were chosen for the testicular transcriptomic analysis because we focused primarily on genes involved in spermatogenesis, and the process of spermatogenesis in this stage was active [19] and presented typical features, such as the emergence of spermatogonia, primary and secondary spermatocytes, spermatids and spermatozoa, and the release into the lobular cavity of many spermatozoa that had developed from spermatids (Figure 2). A total of 25,787 genes were obtained from the two libraries, and 3853 DEGs were screened using a rigorous set of thresholds. Moreover, we identified numerous important functional genes and signaling pathways involved in testicular development and spermatogenesis in *L. crocea* by combining the results of the enrichment analysis, annotations, and manual literature searches. In this study, we mainly focused on the most important functional genes and pathways, including the neuroactive ligand–receptor interaction pathway, GnRH and MAPK signaling pathways, cell cycle pathway, and *dynein*, *kinesin*, *myosin*, *actin*, *hsp*, *sycp2*, *dmrt1*, *spata*, *ddx*, *tdrd*, and *piwi* genes; and we attempted to combine our results with the references and explain the probable role of the genes and pathways in testicular development and spermatogenesis of *L. crocea* in detail.

### 4.1. Neuroactive Ligand–Receptor Interaction Pathway

In the present study, the neuroactive ligand–receptor interaction pathway was the most significantly enriched pathway, with 65 enriched DEGs (Figure 10 and Table S3), which was consistent with that in *Oplegnathus punctatus* [20]. The neuroactive ligand–receptor interaction pathway plays important roles in the reproduction and gonadal development of *Oreochromis niloticus* and *Perca flavescens* [21,22]. Some receptors in the pathway were found to be testis-biased in *L. crocea*. Among these receptors, the follicle-stimulating hormone receptor (*fshr*) and hydroxytryptamine receptor (*htr*) were associated with cell signaling by the hypothalamic–pituitary–gonadal axis (HPG) regulation system and regulated the sex maturity and gonad development [23]. Therefore, we had sound reasons to believe that the neuroactive ligand–receptor interaction pathway may function in both the nervous system and reproduction system to regulate sex hormone secretion.

### 4.2. GnRH and MAPK Signaling Pathway

In vertebrates, the HPG axis plays a significant role in the regulation of reproductive processes. As one of the most crucial elements in the HPG axis, gonadotropin-releasing hormone (GnRH) participates in the regulation of steroidogenesis and gametogenesis by promoting the synthesis and release of follicle-stimulating hormone (FSH) and luteinizing hormone (LH), activating their receptors, and inducing kinds of sex steroid hormones [24]. In the present study, 13 DEGs were assigned to the GnRH pathway, with 5 genes (*mapk10*, *mapkkk2*, *plcb1-like*, *plcb2*, *adcy1*) upregulated and 8 genes (*hbegf*, *plcb1*, *phospholipase d1*, *camk2d*, *ptk2b*, *adcy1*, *cPLA2*, *cPLA2-like*) downregulated in the TES (Table S4), indicating that the GnRH pathway plays a key role in the reproductive system of *L. crocea*, which has also been observed in mammals [25]. Interestingly, we found that *mapk10* was enriched in the GnRH pathway, as mentioned above. According to reports, MAPKs can participate in the transcriptional control of gonadotropin subunits and *GnRHR* genes via GnRH [26]. At the same time, the MAPK signaling pathway was reported to mediate the transit of primary spermatocytes across the blood–testis barrier and facilitate its remodeling during germ cell divisions [27]. MAPKs are transiently activated during mitosis, and their activation has been implicated in the spindle assembly checkpoint and in establishing the timing of unperturbed mitosis [28]. In addition, the MAPK signaling pathway also participated in phosphorylation of proteins in sperm heads [29]. The enrichment of the MAPK signaling pathway in our study (Figure S1) may indicate its role in germ cell divisions and mitosis. Thus, it is reasonable to believe that studies of the interactive network composed of GnRH and MAPK signaling pathway are of great significance for understanding the molecular mechanisms underlying spermatogenesis.

### 4.3. Cell Cycle Pathway

Spermatogenesis, which occurs in male seminiferous tubules and originates from spermatogonia, forms haploid spermatids through extensive cellular remodeling and a series of mitotic and meiotic cell cycles. During these processes, the coordinated activation and inactivation of specific protein kinases are indispensable [30]. In vertebrates, serine/threonine kinases play an essential role in the reorganization of sperm chromatin during spermatogenesis [31]. In our transcriptome dataset, 212 genes were identified according to the Nr annotations with the search term “serine/threonine-protein kinase” (Table S5), including serine/threonine-protein kinase and testis-specific serine/threonine-protein kinases (TSSKs). As a family of post-meiotic kinases expressed in spermatids, TSSKs are essential for spermatogenesis and male fertility [32]. Knockout of the tandemly arranged genes *tssk1* and *tssk2* in mice results in male infertility [33]. In the present study, the RNA-Seq results showed that the 17 serine/threonine-protein kinases genes (rows marked with yellow background in Table S5) were specifically expressed in *L. crocea* testes. Thus, the dataset in our study provides basic clues for further studying the function of these genes in spermatogenesis and testicular development.

In most animals, the combination of cyclin (a regulatory subunit) and cyclin-dependent kinase (CDK, a catalytic subunit) forms a complex of serine/threonine-protein kinase. Reports have shown that CDK and its cyclin partners play an important role in germ cell development and meiosis [34]. In this study, 31 cyclin genes (Table S6) and 27 *cdk* genes (Table S7) were identified in our dataset. Amongst these genes, only *cdk4-like*, *cdk-like 5 isoform x2*, *cdk17-like isoform x2*, *cdk-like 5*, and *cdk2* were specifically expressed in the testes of *L. crocea*. Moreover, the pathway enrichment analysis showed that the cell cycle signaling pathway was significantly enriched (Figure 11). In mammals, the active CDK2/cyclin B1 complex promotes the gap second/mitosis (G2/M) transition in pachytene spermatocytes during meiotic and mitotic cell cycles [34]. None of these genes have been studied extensively in *L. crocea*. The RNA-Seq results here may guide further investigations into the mechanisms underlying reproductive development in male *L. crocea*.

### 4.4. Genes Encoding Microtubule-Based Motor Proteins

In the process of spermatogenesis, the reshaping of the nucleus and the emergence of flagella are typical changes, and microtubules play a vital role in these orderly processes. Microtubules are vital for complicated morphogenesis and the particular packing of organelles. Microtubules function by collaborating with the associated motor proteins, i.e., dynein and kinesin, which are moved in different directions along the microtubules [35], and they support the dynamic force for cytoplasmic proteins and transport vesicles to their destination [35]. Among these, dynein is mostly responsible for the intracellular transportation of the flagella [36]. Microtubule-based cytoskeleton functions in concert with dynein to support the transport of cargoes across the mammalian cell cytosol, including the mitochondria, chromosomes, and other cell organelles [37,38]. Dynein 1 was highly expressed in the TES of this study, and it serves as the engine to support the transport of spermatids and organelles across the seminiferous epithelium during spermatogenesis [39]. In our transcriptome dataset, we identified 17 TES-biased *dynein* subunit transcripts that contained both axonemal and cytoplasmic forms, according to the annotation by Nr (Table S8). The axonemal dyneins are involved in producing flagellar motion, whereas the cytoplasmic dyneins act as molecular motors that move membrane proteins, vesicles, and other cargo in the cell [40]. Relatively speaking, kinesin is more responsible for cellular movement, which includes the spindle apparatus during mitosis and the transport of organelles, protein complexes, mRNA, and some chromatins [41]. In our study, 18 *kinesin* genes (*kifs*), including *kif3a*, *kif3b*, etc., were identified to be highly expressed in the TES (Table S9). The high expression of *kif3a* and *kif3b* in the testis of *L. crocea* was consistent with our previous research results (unpublished results). The findings provide additional insights for further investigations into the molecular mechanisms underlying *kinesins* and *dyneins* in the mitosis of spermatogonia, meiosis of spermatocyte, and intracellular material transportation during spermatogenesis.

### 4.5. Actin Cytoskeleton and Myosins

During spermiogenesis, the round spermatids differentiate into well-shaped spermatozoa through cellular remodeling and nuclear morphogenesis, in which dramatic changes occur in the cytoskeletal structures [42]. As a key cytoskeletal structure, actin is believed to function in spermiogenesis [43,44,45], and mutations of actin lead to abnormal sperm heads [46]. F-actin (filamentous actin) is contained in the manchette (a structure consisting of a perinuclear ring and parallel cytoplasmic microtubules) presumably and serves as one cytoskeletal track to facilitate the transport of proteins and proacrosomal vesicle cargo during spermiogenesis [47]. In this study, we detected 310 gene transcripts encoding actin, actin-binding, and actin-related proteins that were likely to contribute to the regulation of the actin cytoskeleton pathway (Table S10). Their roles in the spermiogenesis of *L. crocea* needs further research.

Among the above 310 genes in the regulation of the actin cytoskeleton pathway, 12 transcripts annotated as myosin regulatory light chain and myosin light chain kinases were found (rows marked with a yellow background in Table S10). Myosins constitute a superfamily of the actin-based molecular motors that translocate along microfilaments in an ATP-dependent manner. Myosins were found to play an important role in spindle assembly and positioning, karyokinesis and cytokinesis, acrosomal formation, nuclear morphogenesis, mitochondrial translocation, and spermatid differentiation [42]. Each type of myosin plays different but necessary roles during spermatogenesis. Myosin X is responsible for cytokinesis, which regulates the spindle assembly and chromosome separation in mitosis and meiosis during spermatogenesis [48]. Myosin V has been proved to mediate vesicle trafficking, acrosome formation, intramanchette transport, and nuclear shaping during spermatogenesis in mammals [49]. Myosin VI is involved in maintaining the actin cone organization [50] and stabilizing the actin network [51]. Myosin VII may also function in the development of Sertoli cells and germ cells [52]. In our study, 12 *myosin* genes were highly expressed in the TES, including *myosin-Va*, *myosin-7B*, *myosin-10*, etc., (data not shown), suggesting that *myosins* are essential in the process of spermatogenesis in *L. crocea*.

### 4.6. Heat Shock Protein Genes (HSPs)

Heat shock proteins (HSPs) play a crucial role in cell defense against adverse environmental conditions, and effectively maintain cell survival. Furthermore, HSPs are documented to be potentially important in testis [53]. The *hsp70*, *hsp60*, and *hsp90* family genes are the most abundantly expressed in testis [53]. HSPs were found to be involved in apoptosis/anti-apoptosis in spermatogenic cells [54]. Specifically, HSP70 plays an important role in spermatogenesis and sperm maturation in several species, such as mice [55] and chicken [56]. Dysregulation of *hsp70* is positively correlated with infertility in human sperm samples [57]. In this study, 31 *hsps* were detected in our transcriptome dataset (Table S11), including small *hsps*, *hsp40*, *hsp60*, *hsp70*, and *hsp90* families, which is similar to the results of studies in the male *Bactrocera dorsalis* [32], *Eriocheir sinensis* [58], and *Oryctolagus cuniculus* [53], which indicates the high conservation of *hsp* gene families among organisms. Amongst the 31 *hsp* genes, only *hsp70-1*, *hsp70-4-like*, *hsp70-4l*, and *dnaJ homologue subfamily A member 1* were highly expressed in the TES. There is little research on the function of *L. crocea* HSPs in spermatogenesis and testicular development. Therefore, further experimentation is required to investigate the functions of these HSPs in spermatogenesis in *L. crocea*.

### 4.7. Synaptonemal Complex Protein 2 Gene (Sycp2)

SYCP2, a constituent element of axial/lateral elements (AEs/LEs), was reported to be essential for chromosome synapsis in male meiosis and the formation of normal AEs. The deletion of *sycp2* resulted in spermatocyte apoptosis [59]. In the present transcriptome dataset, *sycp2* was found to be highly expressed in the testes. We speculate that it might play a similar role in the testis of *L. crocea*.

### 4.8. Doublesex- and Mab-3-Related Transcription Factor 1 (Dmrt1)

DMRT1 was identified to regulate germline maintenance and gonadal development through estrogen/androgen signaling [60]. Previous research showed that DMRT1 may play an essential role in male development in *Cynoglossus semilaevis* [61]. The knockdown of the *dmrt1* gene in early chicken embryos can result in the feminization of male gonads [62]. Disruption of *dmrt1* in male *O. niloticus* resulted in significant testicular regression [60]. In addition, its expression is correlated with the proliferation of spermatogonia in *T. rubripes* [63]. According to our results, *dmrt1* is a testis-specific gene in *L. crocea*, suggesting that *dmrt1* may be a key regulator in the testicular development of *L. crocea*.

### 4.9. Spermatogenesis-Associated Genes (Spatas)

*Spatas* were reported to be implicated in the regulation of apoptosis during spermatogenesis [64]. *Spata4* was previously reported to be a testis-specific gene in animals, ranging from birds to mammals [65,66]. In the present study, the higher expression level of *spata4* in the TES than that in the PMT (Table S12) indicates that its function in *L. crocea* is consistent with that in humans and chickens [65,66]. In addition to *spata4*, a testis-biased expression profile was observed for other *spatas* genes, such as *spata1*, *spata6*, *spata7*, *spata17*, *spata20*, and *spata22*, whose expression levels in the TES were all higher than that in the PMT (Table S12), suggesting the potential roles of these gene during spermatogenesis in *L. crocea*.

### 4.10. DEAD-Box Helicases (Ddxs)

DDXs are a group of motor proteins with significant roles in regulating the reproductive-related genes by modulating mRNA structures [67]. DDXs were reported to be involved in embryogenesis, spermatogenesis, cellular growth, division, and maturation [68]. Of the RNA helicases, DDX4 (also known as VASA) has been reported to be involved in the gametogenesis of some fishes, such as *Danio rerio* [69] and *I. punctatus* [70]. Disruption of *vasa* led to germ cell specification failure and defects in germ cell development in mice [71]. In the present study, *Ddx4* exhibited a high level of expression in the *L. crocea* testis and was identified as a testis-biased gene. Rolland et al. performed in situ hybridization and detected a very strong signal for *ddx4* in rainbow trout testis [72], which is consistent with our results. In addition to *ddx4*, *ddx19b* and *ddx43* were also highly expressed in the testis. In *L. crocea*, the exact functions of these *ddx* genes in the reproductive system are not clear, but are certainly worthy of additional studies.

### 4.11. Tudor Domain-Containing Protein Genes (Tdrds) and Piwis

*Tdrd1*, which is essential for spermatogenesis, was found to be a testis-specific gene in this study, and it was previously reported to be preferentially expressed in murine testis [73] and to play a significant role in spermatogenesis [74]. In addition to *tdrd1*, other *tdrd* genes were highly expressed in the TES, namely, *tdrd5*, *tdrd6-like*, *tdrd9*, *tdrd12*, and *tdrd15-like*. Each member of the *tdrd* gene family performs a distinct function at different differentiation stages of spermatogenesis. TDRD7, whose gene was identified in this study but without a significant difference between the TES and the PMT, was demonstrated to play a crucial role during early spermatid differentiation [75]. Furthermore, TDRDs were reported to be physiological binding partners of Piwi family proteins, and they regulate the processes of spermatogenesis through transcriptional and post-transcriptional regulation [73,76]. In this study, we also identified two *piwi* family genes, namely *piwil1* and *piwil2*, as testis-biased genes. Our findings show that the high expression of both *piwis* and *tdrds* in the TES indicate that they may work cooperatively in the regulation of spermatogenesis. Further investigations are needed to clarify the functions of these genes in *L. crocea*.

## 5. Conclusions

A comparative transcriptome analysis was performed, and we found a considerable amount of testis development-related and spermatogenesis-related genes and pathways in *L. crocea*, including the neuroactive ligand–receptor interaction pathway, GnRH and MAPK signaling pathways, cell cycle pathway, and the *dynein*, *kinesin*, *myosin*, *actin*, *hsp*, *sycp2*, *dmrt1*, *spata*, *ddx*, *tdrd*, and *piwi* genes. These genes and pathways showed significant similarities to that in other fishes, birds, and mammals, suggesting that the mechanisms underlying the male reproductive system in fish may be conserved in vertebrates. This transcriptome dataset will enrich the genomic information for *L. crocea* and pave the way towards an explanation of the molecular mechanisms underlying testicular development and spermatogenesis in this species. The full-length amplification of candidate genes and the validation of their functions will be our next research topic.

## 6. Data Accessibility

All reads were deposited in the Short Read Archive (SRA) of the National Center for Biotechnology Information (NCBI) under the accession numbers SRP148410 for the TES and SRP148493 for the PMT.

## Figures and Tables

**Table 1:** MIxS information for the transcriptome of *L. crocea*.

Item	Description
Investigation type	Eukaryote
Project name	Transcriptome for *L. crocea*
Collection date	May 2016
Lat_lon	29°86′ N, 121°56′ E
Geo loc name	Ningbo, China
Environment	Marine water
Biotic_relationship	Free-living
Trophic level	Heterotroph
Temp	21 °C
Salinity	24 PSU
Estimated size	16.35 Gb
Sequencing meth	Illumina HiSeq^TM^ 2500
Mapping software	TopHat2
Annotation source	Nr/Nt/Swiss-Prot/KEGG/COG/GO
BioProject ID of raw reads	PRJNA471154 for TES/PRJNA471574 for PMT
Accession number of raw reads	SRP148410 for TES/SRP148493 for PMT

**Table 2:** Primers used for qPCR.

Gene ID	Annotation	Primer	Sequence (5′→3′)
gene15399	*tex9*	F	GCTGTAGACGACTCGGCTGACTT
		R	TGAGCATCTGAAACGCCTGATCCT
gene14348	*tex36*	F	GCAAGGAGTTGTCACACTGGCATC
		R	TCCATCGTGGCACAGGCAGAAG
gene2626	*tex11*	F	TCGGTGAAGTCTCTGCTCTGGAAG
		R	GGACGCCCTCTGTTGGATTCTCA
gene21265	*tcte1*	F	TGATCGGAGACAGAGGAGCCAGAG
		R	CGGTTGAGACGCAGGTTGAGTGA
gene20395	*stpg2*	F	GCAGTCTCCAGAACCGCTCCAA
		R	CAGTGTGTCCTCCTCGTAGCCAAA
gene3104	*spata7*	F	CTGAGGATGAGTCCAACGGCACAT
		R	AGATTTCCCGCCTTCTGGTGAGAC
gene26146	*spata20*	F	GTTCCTGGACGACTACGCCTTCAT
		R	TGGACGCCGACACTGAGTTAGC
gene26767	*spag5*	F	GGACATCCAGCAAGCCAATGACAG
		R	CCTCGCCAACTCGTTCTCCATCT
gene26247	*spag17*	F	CCAGACGAGGAGGAGGACAGAGAA
		R	TTCAGGATGGTGATGCCGAACTCA
gene16701	*pabp1l*	F	AGTCCGCTAATGGAGGCTCTGTC
		R	AGTGGTGGTCCTTGTGGTTGATGT
gene10808	*nme5h*	F	TCCACGGCAGCGAGTCATTTCAT
		R	TCAGCCAGTCAGCAAGCCAGATAC
gene21216	*meig1*	F	ACAACTCCAAGCCGAAGTCCATGA
		R	TTGACATCACGGTCCAGGCACTC
gene13149	*lhcgrl*	F	TGGCATCAAGGAGGTGGCAAGT
		R	TGGTAGGCGGACTCTGCGATCA
gene1213	*hsp40a1*	F	AGGTCGTGGGAGTCGGAAAGGA
		R	TGTGGACACTTGCTGGACCATACC
gene17675	*ggn*	F	GCTGAAGTGCCACCTGAGTCACA
		R	GCCGCTGTTGTATTGCTGCTCTG
XM_019257255.1	*β-actin*	F	CTGTCCCTGTATGCCTCTGGTC
		R	CTTGATGTCACGCACGATTTCC

**Table 3:** Summary of *L. crocea* transcriptome data.

Samples	TES	PMT
Clean reads	26,201,689	28,786,700
Clean bases (Gb)	7.8	8.5
GC percentage	50.73%	50.16%
% ≥ Q20	93.38%	93.64%
% ≥ Q30	85.81%	86.27%
Total mapped	33,390,310 (63.72%)	38,470,859 (66.82%)
Uniquely mapped	30,802,638 (58.78%)	35,380,000 (61.45%)

**Table 4:** Biological processes involved in spermatogenesis and testicular development.

GO.ID	The Third-Level Functional Categories	All Gene Number	DEG Number	*KS* ^a^
GO:0001539	cilium or flagellum-dependent cell motility	18	12	4.60E-06
GO:0007018	microtubule-based movement	61	31	7.90E-05
GO:0007283	spermatogenesis	20	11	0.00017
GO:0003006	developmental process involved in reproduction	70	23	0.0035
GO:0048515	spermatid differentiation	10	5	0.00452
GO:0007126	meiotic nuclear division	22	10	0.00474
GO:0044702	single organism reproductive process	89	30	0.00857
GO:0071695	anatomical structure maturation	6	1	0.00991
GO:0007286	spermatid development	9	4	0.01166
GO:0046903	secretion	64	8	0.01212
GO:0090068	positive regulation of cell cycle process	12	2	0.01577
GO:0033143	regulation of intracellular steroid hormone receptor signaling pathway	6	1	0.01586
GO:0007281	germ cell development	29	11	0.01627
GO:0035265	organ growth	11	4	0.01688
GO:0000910	cytokinesis	25	7	0.018
GO:0051495	positive regulation of cytoskeleton organization	23	4	0.01847
GO:0048729	tissue morphogenesis	289	50	0.01997
GO:0002009	morphogenesis of an epithelium	236	38	0.02012
GO:0051653	spindle localization	12	1	0.02114
GO:0048608	reproductive structure development	38	9	0.02247
GO:0061458	reproductive system development	38	9	0.02247
GO:0007010	cytoskeleton organization	223	50	0.02305
GO:0045010	actin nucleation	14	4	0.02346
GO:0007548	sex differentiation	33	9	0.02645
GO:0060070	canonical Wnt signaling pathway	77	12	0.03034
GO:0008406	gonad development	28	8	0.03043
GO:0060029	convergent extension involved in organogenesis	12	1	0.03087
GO:0051493	regulation of cytoskeleton organization	63	11	0.03124
GO:0007017	microtubule-based process	170	58	0.03224
GO:0030029	actin filament-based process	132	30	0.0363
GO:0046330	positive regulation of JNK cascade	11	1	0.04252
GO:0030036	actin cytoskeleton organization	130	30	0.04805

^a^*KS* < 0.05 indicates statistical significance of enrichment.

## Supplementary Materials

The following are available online at https://www.mdpi.com/2073-4425/10/12/958/s1, Table S1. Functional annotation of differentially expressed genes (DEGs) according to the COG, GO, KEGG, KOG, Pfam, SWISS-PROT, eggNOG, and Nr databases; Table S2. Summary of GO annotations for testes-biased genes and somatic-biased genes; Table S3. DEG annotations to 152 KEGG pathways; Table S4. DEGs involved in the GnRH pathway; Figure S1. DEGs enriched in the MAPK pathway; Table S5. Transcript annotations in Nr with the search term “serine/threonine-protein kinase”; Table S6. Transcript annotations in Nr with the search term “cyclin”; Table S7. Transcript annotations in Nr with the search term “cyclin-dependent kinase”; Table S8. Annotation of TES-biased dyneins; Table S9. Annotation of TES-biased kinesins; Table S10. Genes involved in the actin cytoskeleton pathway; Table S11. Transcript annotations in Nr with the search term “hsp”; Table S12. Spata genes in the TES-biased genes.
