# Supplementary material for: Transcriptome Sequencing Reveals the Traits of Spermatogenesis and Testicular Development in Large Yellow Croaker (Larimichthys crocea)
Source: Genes (Basel). 2019 Nov 21;10(12):958. doi: 10.3390/genes10120958 (PMC6947352; doi:10.3390/genes10120958)
Supplement: Supplementary file 1 [file genes-10-00958-s001.zip › Table S2.docx]

Table 4. Summary of GO annotations for testes-biased genes and somatic-biased genes

| **Category** | **Testes-biased genes** | | **Somatic-biased genes** |
| --- | --- | --- | --- |
| **biological process** | | **634 (81.70%)** | **821(88.18%)** |
| cellular process | | 429 | 400 |
| single-organism process | | 335 | 456 |
| metabolic process | | 265 | 352 |
| biological regulation | | 228 | 298 |
| developmental process | | 133 | 147 |
| multicellular organismal process | | 124 | 156 |
| response to stimulus | | 119 | 199 |
| localization | | 109 | 150 |
| signaling | | 79 | 125 |
| cellular component organization or biogenesis | | 76 | 49 |
| reproduction | | 25 | 9 |
| reproductive process | | 25 | 9 |
| locomotion | | 24 | 27 |
| multi-organism process | | 13 | 12 |
| biological adhesion | | 9 | 17 |
| immune system process | | 7 | 56 |
| behavior | | 5 | 0 |
| growth | | 2 | 8 |
| rhythmic process | | 1 | 0 |
| detoxification | | 0 | 4 |
| **cellular component** | | **438 (56.44%)** | **571(61.33%)** |
| cell | | 333 | 276 |
| organelle | | 237 | 135 |
| membrane | | 160 | 259 |
| organelle part | | 110 | 61 |
| membrane part | | 108 | 194 |
| supramolecular complex | | 30 | 20 |
| extracellular region | | 14 | 94 |
| cell junction | | 10 | 11 |
| synapse | | 7 | 2 |
| synapse part | | 6 | 1 |
| nucleoid | | 1 | 0 |
| cell part | | 0 | 276 |
| extracellular region part | | 0 | 32 |
| macromolecular complex | | 0 | 73 |
| membrane-enclosed lumen | | 0 | 5 |
| **molecular function** | | **589 (75.90%)** | **779(83.67%)** |
| binding | | 410 | 471 |
| transporter activity | | 46 | 96 |
| nucleic acid binding transcription factor activity | | 45 | 47 |
| signal transducer activity | | 35 | 88 |
| molecular function regulator | | 17 | 43 |
| structural molecule activity | | 15 | 16 |
| antioxidant activity | | 1 | 4 |
| transcription factor activity, protein binding | | 1 | 3 |
| catalytic activity | | 0 | 373 |
| chemoattractant activity | | 0 | 2 |
| electron carrier activity | | 0 | 18 |
| molecular transducer activity | | 0 | 92 |
